# Necrosis- and apoptosis-related Met cleavages have divergent functional consequences

**DOI:** 10.1038/cddis.2015.132

**Published:** 2015-05-21

**Authors:** R Montagne, M Berbon, L Doublet, N Debreuck, A Baranzelli, H Drobecq, C Leroy, N Delhem, H Porte, M-C Copin, E Dansin, A Furlan, D Tulasne

**Affiliations:** 1 CNRS UMR 8161, Institut de Biologie de Lille–Institut Pasteur de Lille, Université de Lille, SIRIC ONCOLille, Lille 59021, France; 2 Département de Cancérologie Générale, CLCC Oscar Lambret, Université de Lille, 3 rue Fréderic Combemale, Lille 59020, France; 3 Service de Chirurgie Thoracique, Centre Hospitalier Régional Universitaire de Lille, Université de Lille, Lille, France; 4 Institut de Pathologie, Centre Hospitalier Régional Universitaire de Lille, Université de Lille, Avenue Oscar Lambret, Lille 59037, France

## Abstract

Upon activation by its ligand hepatocyte growth factor/scatter factor, the receptor tyrosine kinase Met promotes survival, proliferation, and migration of epithelial cells during embryogenesis. Deregulated Met signaling can also promote cancer progression and metastasis. Met belongs to the functional family of dependence receptors whose activity switches from pro-survival to pro-apoptotic during apoptosis upon caspase cleavage. Although apoptosis resistance is a hallmark of cancer cells, some remain sensitive to other cell death processes, including necrosis induced by calcium stress. The role and fate of Met during necrotic cell death are unknown. Following treatment with calcium ionophores, cell lines and primary cells undergo necrosis, and the full-length Met receptor is efficiently degraded. This degradation is achieved by double cleavage of Met in its extracellular domain by a metalloprotease of the A disintegrin and metalloproteinase (ADAM) family and in its intracellular domain by calpains (calcium-dependent proteases). These cleavages separate the Met extracellular region from its kinase domain, thus preventing Met activity and its potential pro-survival activity. Although the intracellular fragment is very similar to the fragment generated by caspases, it displays no pro-apoptotic property, likely because of the presence of the last few amino acids of Met, known to inhibit this pro-apoptotic function. The fragments identified here are observed in lung tumors overexpressing the Met receptor, along with fragments previously identified, suggesting that proteolytic cleavages of Met are involved in its degradation in tumor tissues. Thus, Met is a modulator of necrosis, able to protect cells when activated by its ligand but efficiently degraded by proteolysis when this process is engaged.

Met is a receptor tyrosine kinase expressed predominantly by epithelial cells and activated by its stromal ligand, hepatocyte growth factor/scatter factor (HGF/SF). Met activation stimulates a biological program called invasive growth,^1^ involving survival, proliferation, invasion, and morphogenesis of epithelial cells. Ligand-stimulated Met acts, furthermore, as an angiogenic and neurotrophic factor.^2, 3^ HGF/SF and Met are essential to several steps of embryogenesis, experiments on transgenic mice having shown that they are necessary for formation of the placenta, liver, limb muscle, neurons, and lung airspace.^4, 5, 6, 7, 8^ In adults, HGF/SF and Met promote regeneration of several organs, including the liver, kidneys, and thymus.^9, 10, 11, 12, 13^

Aberrant Met and HGF/SF signaling contributes to promoting tumorigenesis and metastasis (for review see Furlan *et al.*).^14^ A direct link between Met and cancer has been evidenced by observation of Met germinal mutations linked to hereditary papillary renal carcinoma.^15^ Met and/or HGF/SF are/is also overexpressed in several human cancers.^16^ Given its important oncogenic activity, Met is the target of many therapeutic agents currently under clinical investigation.^17^

Downregulation of Met following its activation by HGF/SF is an important negative regulatory mechanism preventing receptor overactivation. We have previously shown that Met expression and activity are also controlled by proteolytic cleavages. Under steady-state conditions, Met is processed by PS-RIP (presenilin-regulated intramembrane proteolysis).^18, 19^ This process involves cleavage of Met within its extracellular juxtamembrane domain by A disintegrin and metalloproteinase (ADAM)-10,^20^ generating a soluble N-terminal fragment (Met-NTF), which is released into the extracellular space, and a membrane-anchored C-terminal Met fragment (Met-CTF). The latter is in turn efficiently degraded by the lysosome and by further *γ*-secretase cleavages. Constitutive degradation of the Met receptor by PS-RIP contributes to regulating its half-life.

Under apoptotic conditions, Met is cleaved by caspases^21^ within its C-terminal tail and its intracellular juxtamembrane domain. These cleavages remove the C-terminal tail of Met and separate the extracellular ligand-binding domain from the intracellular kinase domain. The generated 40-kDa intracellular fragment, previously called ‘p40Met' and here called p40Met^caspase^, can increase cell death by promoting mitochondrial permeabilization.^22, 23^ Removal of the C-terminal tail of Met is required for the efficient pro-apoptotic action of the fragment. This pro-apoptotic function of Met makes it a member of the dependence receptor family.^24^ Met cleavages are illustrated in Figure 6a.

Although the mechanisms underlying apoptosis have been studied extensively, necrosis has only recently been described as a regulated cell death mechanism.^25^ Necrosis is an adenosine triphosphate (ATP)-independent cell death mechanism featuring early plasma membrane and organelle disruption. Many pathways can lead to cell necrosis, including calcium overload. This type of cell stress has been amply described in the nervous system, where an increase in intracellular calcium results in neuronal injury and neurodegenerative diseases. In many other cell types, calcium ionophores such as ionomycin can induce rapid necrosis. An increase in intracellular calcium triggers activation of several proteases, including calpains and cathepsins.^26, 27, 28^ Calpains are calcium-dependent proteases capable of cleaving multiple substrates and involved in regulating various cellular processes, including migration, autophagy, apoptosis, and necrosis. Interestingly, the effector role of calpains during necrosis is reminiscent of the function of caspases during apoptosis. Caspases are directly involved in morphological changes observed during apoptosis, while calpains can cleave cytoskeletal proteins such as spectrin and tubulin, thus favoring dismantling of cell structure during necrosis.^29, 30, 31^

Although apoptosis resistance is a hallmark of many cancer cells,^32^ some such cells remain sensitive to other cell death processes, including necrosis.^33^ Thus, a better understanding of the mechanisms underlying necrosis is important, as it could help to elaborate novel therapeutic strategies. Here we show that calcium stress induced by calcium ionophores triggers Met degradation during necrotic cell death. This loss of Met receptor occurs early during the process and is mediated by Met cleavages: by calpains in its intracellular part and by metalloproteases in its extracellular part. These cleavages generate an extracellular fragment and an intracellular fragment with a molecular weight close to that of p40Met^caspase^.

## Results

### Calcium-stress-induced necrosis triggers Met degradation

**Figure 1:**
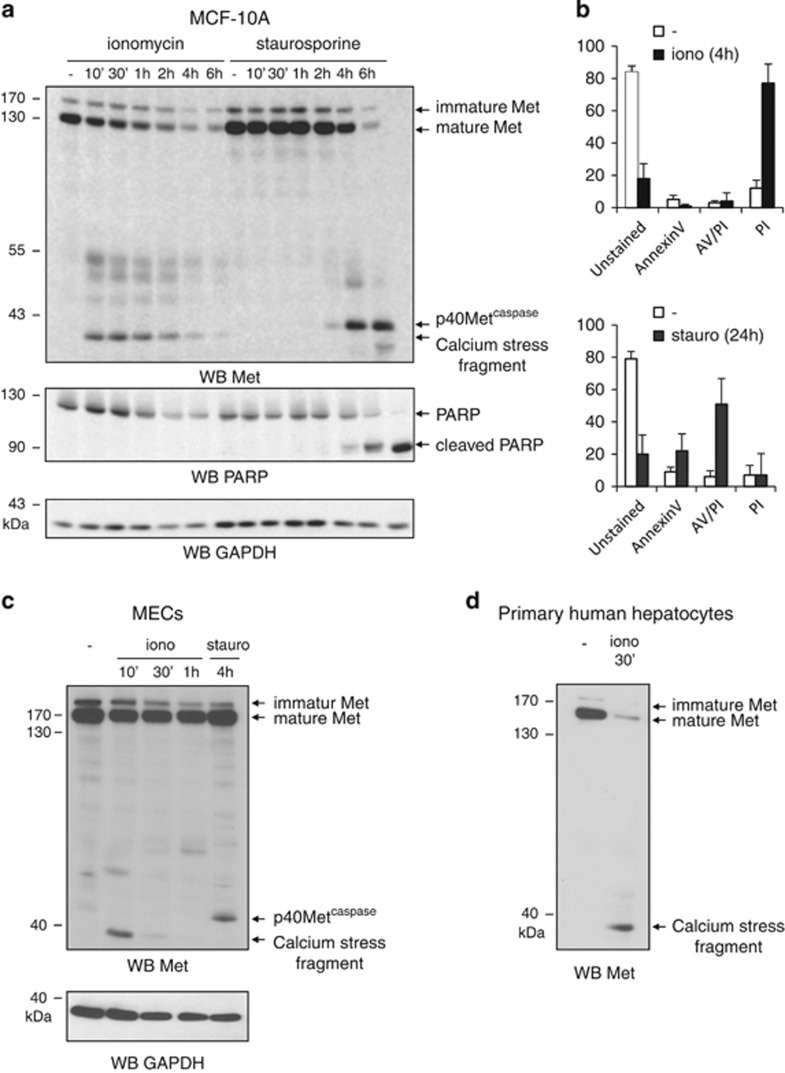
Ionomycin induces necrotic cell death and generation of a 40-kDa Met fragment. (**a**) MCF-10A cells were grown for 24 h, serum starved overnight, and treated with 1 *μ*M ionomycin (iono) or 1 *μ*M staurosporine (stauro) for the indicated time. (**b**) MCF-10 A cells were treated with 1 *μ*M ionomycin or 1 *μ*M staurosporine then stained with Annexin V-fluorescein isothiocyanate (FITC) and PI. Percentages of unstained cells and of cells stained with Annexin V, PI, or both (AV/PI) are shown. Mouse mammary epithelial cells (MECs) (**c**) or human primary hepatocytes (**d**) were treated with 1 *μ*M ionomycin or 1 *μ*M staurosporine for the indicated time. (**a**, **c** and **d**) Cell lysates were analyzed by western blotting with an antibody directed against the kinase domain of human Met, against PARP to assess caspase activation, or against GAPDH to assess loading. Arrows indicate full-length Met, p40Met^caspase^, and the calcium-stress-induced fragment

In order to investigate the fate of Met during necrosis, we administered different pharmacological drugs known to induce either necrosis or apoptosis. Treatment of MCF-10A epithelial cells with staurosporine triggered cell death with the distinctive features of apoptosis, including cleavage of the caspase substrate poly(ADP-ribose) polymerase (PARP), Annexin V staining, along with degradation of full-length Met, and production of the pro-apoptotic fragment p40Met^caspase^ (Figures 1a and b). In contrast, treatment with the calcium ionophore ionomycin induced, after a few hours, cell death without PARP cleavage or any significant phosphatidylserine externalization. Instead, the cells displayed sustained propidium iodide (PI) staining, suggesting early plasma membrane disruption consistent with known necrotic cell death induced by calcium ionophores (Figure 1b). Ionomycin treatment also induced early degradation of Met and production of a fragment of ~40 kDa, only slightly different in molecular weight from p40Met^caspase^ (Figure 1a). Faint bands were also observed ~55 kDa, reminiscent of the Met-CTF fragments generated by PS-RIP.^18, 19^ Ionomycin treatment was found to cause similar Met degradation and 40-kDa fragment generation in MDA-MB231 breast cancer cells and Met-overexpressing GTL-16 gastric cancer cells (Supplementary Figures S1A and B), and also in primary cultures of mouse mammary epithelial cells and in human primary hepatocytes (Figures 1c and d). Generation of the 40-kDa fragment was observed from 100 nM ionomycin upward, with maximal generation at 1 *μ*M, associated with degradation of full-length Met (Supplementary Figure S1C). Ionomycin treatment in calcium-free medium failed to induce Met degradation (Supplementary Figure S1D), while treatment of MCF-10A cells with A23187, another calcium ionophore, caused a decrease in full-length Met and generation of the 40-kDa fragment (Supplementary Figure S1E).

**Figure 2:**
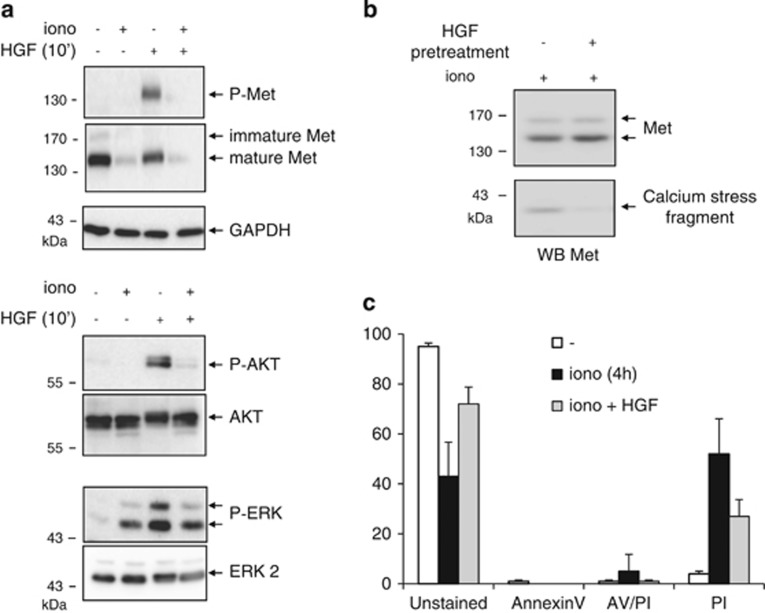
Calcium-stress-induced Met degradation impairs Met survival signaling. (**a**) MCF-10 A cells were grown for 24 h, serum starved overnight, treated for 1 h with 1 *μ*M ionomycin (iono), and then stimulated or not 10 min with 10 ng/ml HGF/SF. (**b** and **c**) MCF-10 A cells were grown for 24 h, serum starved overnight in the presence of vehicle or 10 ng/ml HGF/SF, then treated for 1 h with 1 *μ*M ionomycin and (**b**) analyzed by western blotting or (**c**) stained with Annexin V-fluorescein isothiocyanate (FITC) (AV) and PI. (**a** and **b**) Cell lysates were analyzed by western blotting with antibodies directed against the kinase domain of human Met, AKT, ERK, or the phosphorylated form thereof (P-Met, P-AKT, or P-ERK) and against GAPDH to assess loading. Arrows indicate the positions of the respective detected proteins

During calcium-induced necrosis, consistently with the observed efficient degradation of Met, HGF/SF stimulation of ionomycin-pretreated cells failed to activate Met and its downstream signaling proteins ERK (extracellular signal-regulated kinase) and AKT (Figure 2a). It is worth noting that ionomycin treatment did cause ERK activation, but that HGF/SF was unable to induce its further stimulation. To evaluate the survival response potentially induced by Met, HGF/SF-pretreated cells were incubated with ionomycin. HGF/SF was found to reduce Met degradation and generation of the 40-kDa fragment, concomitantly with a decrease in PI staining (Figures 2b and c).

### Calpains perform intracellular Met cleavage during necrosis

**Figure 3:**
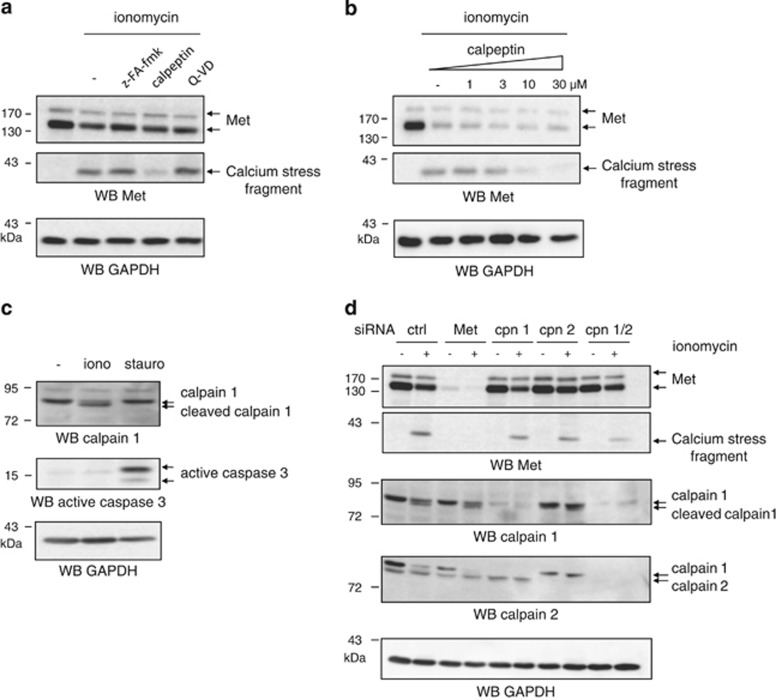
Ionomycin induces intracellular Met cleavage by calpains. MCF-10A cells were grown for 24 h, serum starved and treated with (**a**) 20 *μ*M cathepsin inhibitor Z-FA-FMK, calpain inhibitor calpeptin, or pan-caspase inhibitor QVD or (**b**) increasing concentrations of calpeptin. They were then treated for 1 h with 1 *μ*M ionomycin (iono). (**c**) MCF-10A cells were grown for 24 h, serum starved, and treated for 1 h with 1 *μ*M ionomycin or for 6 h with 1 *μ*M staurosporine (stauro). (**d**) MCF-10A cells were transfected with the same amount of siRNA. The siRNAs tested were as follows: control siRNAs, siRNAs targeting Met or calpain 1 or calpain 2, or a mix of siRNAs targeting calpain 1 and calpain 2. On the next day, they were serum starved overnight and treated for 1 h with 1 *μ*M ionomycin. (**a**–**d**) Cell lysates were analyzed by western blotting with an antibody directed against either the kinase domain of human Met, calpain 1, calpain 2, cleaved caspase 3, or GAPDH to assess loading. Arrows indicate the respective positions of the detected proteins and their cleaved forms. Sequential reprobing with calpain 1 and calpain 2 resulted in detection of both proteases (**d**)

To see whether the 40-kDa fragment observed during calcium stress is generated by proteolytic cleavage, we incubated epithelial cells with various protease inhibitors before ionomycin treatment. In contrast to caspase and cathepsin inhibitors (Figure 3a), the calpain inhibitor calpeptin was found to decrease production of the 40-kDa fragment in a dose-dependent manner (Figures 3a and b). Activation of calpains during calcium stress was confirmed by calpain 1 autolysis, detected by generation of a cleaved form (Figures 3c and d and Supplementary Figure S1C). Met-receptor-targeting ribonucleic acid (RNA) interference led to loss of the calcium stress fragment, confirming that it is a fragment of the Met receptor. RNA interference targeting calpains 1 and 2 (the two best-characterized ubiquitous calpain isoforms) decreased (but did not totally prevent) production of the calcium stress fragment, indicating an involvement of these two proteases (Figure 3d). Neither calpain inhibitor treatment nor RNA interference targeting calpains 1 and 2 was found to restore detection of full-length Met.

### p40Met^calpain^ does not exert pro-apoptotic action

**Figure 4:**
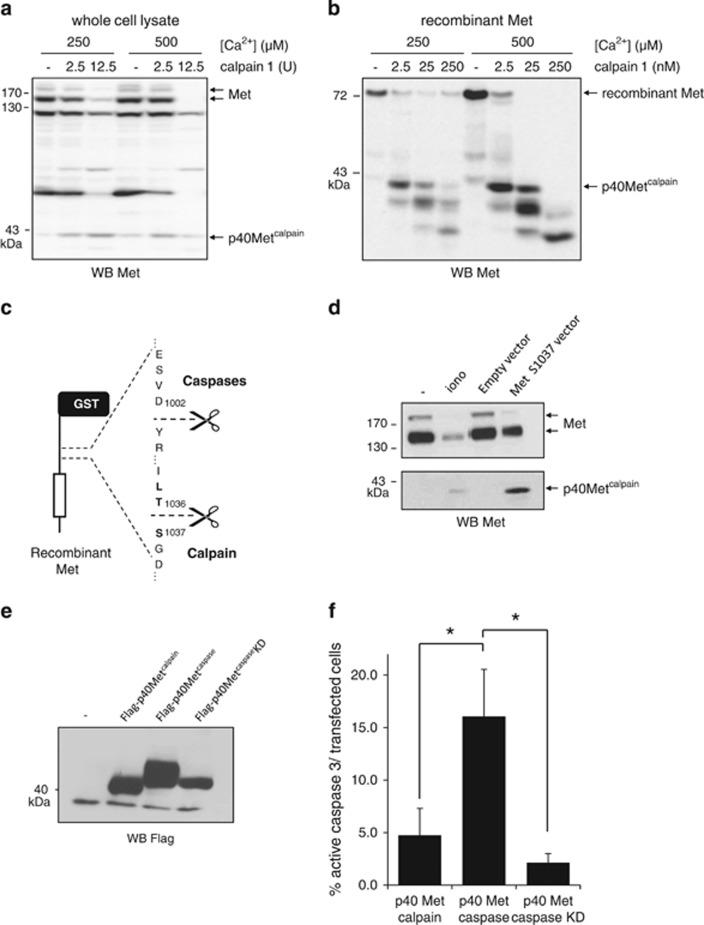
Reconstitution of p40Met^calpain^ and evaluation of its apoptotic properties. (**a**) Equal amounts of extract obtained by lysing MCF-10A cells in LR buffer or (**b**) 5 ng purified recombinant intracellular Met domain were incubated with the indicated concentration of CaCl_2_ and purified calpain 1 for 30 min. (**c**) Schematic representation of the caspase and calpain cleavage sites in the juxtamembrane domain of Met. (**d**) MCF-10 A cells were grown for 24 h and either serum starved and treated for 1 h with 1 *μ*M ionomycin (iono) or transfected with a vector expressing the Met S1037 variant. (**a**, **b** and **d**) Cell lysates were analyzed by western blotting with an antibody directed against the kinase domain of human Met. Arrows indicate the positions of Met, recombinant Met, and p40Met^calpain^. (**e**) HEK cells were transfected with empty vector or a vector expressing Flag-tagged versions of p40Met^caspase^, p40Met^caspase^ K1108A (kinase dead, KD), or p40Met^calpain^. Twenty-four hours later, the cells were lysed and lysates were analyzed by western blotting with an antibody directed against Flag. (**f**) MCF-10A cells were cultured on glass coverslips and transfected with the indicated Flag-p40Met. Twenty-four hours later, the cells were fixed and stained with antibodies against Flag and active caspase 3. Flag-positive cells (expressing p40 Met) and displaying active caspase 3 staining were counted. About 1000 flag-positive cells were counted per well and percentage was calculated (*n*=3; ±S.D.). The *P*-value of Student's *t*-test is shown (*P<0.05)

To confirm the involvement of calpains, we incubated purified calpain 1 with cell extracts or with the recombinant intracellular domain of Met. In the presence of calcium, cleavage of full-length Met in MCF-10A lysate by purified calpain 1 generated a Met-CTF fragment of the same size as the calcium stress fragment (Figure 4a). Similar proteolytic processing of the recombinant intracellular Met domain was observed in the presence of 2.5 nM calpain, with generation of a major 40-kDa fragment. Further cleavage to smaller fragments was observed with excess protease (Figure 4b). By analogy to p40Met^caspase^, this novel fragment was called p40Met^calpain^.

Despite many attempts to predict consensual calpain cleavage sites, it seems likely that these proteases recognize secondary and ternary structures, making cleavage sites difficult to identify.^34, 35^ To determine the calpain cleavage sequence, we analyzed the *in vitro* Met cleavage product by mass spectrometry. AspN digestion followed by mass spectrometry revealed that the first N-terminal peptide begins at amino acid D1041, suggesting that cleavage occurs before this sequence (Supplementary Figures S2A and B). Mass spectrometry also showed that p40Met^calpain^ still includes the last amino acids of Met. A specific antibody targeting the C-terminal tail of Met detected p40Met^calpain^ but failed to detect p40Met^caspase^, demonstrating that calpain processing of Met preserves its C-terminal end (Supplementary Figure S3). Analysis of the putative calpain cleavage region with the SitePrediction tool^34^ identified a potential cleavage site between residues T1036 and S1037 (Figure 4c). Therefore, we produced in transfected cells expressing an appropriate construct a version of Met starting at residue S1037 and ending at the natural stop codon. Western blot analysis showed that this fragment has the same molecular weight as endogenous p40Met^calpain^ (Figure 4d).

We have previously demonstrated that loss of the C-terminal tail of Met is an important step in reshaping Met into a pro-apoptotic factor.^22, 23^ Because the p40Met^calpain^ sequence is quite similar to p40Met^caspase^ but retains the C-terminal tail, we wondered whether p40Met^calpain^ shares the ability of p40Met^caspase^ to induce cell death. When epithelial cells were transfected with a construct encoding either Flag-p40Met^caspase^, Flag-p40Met^calpain^, or a non-apoptotic version of p40Met^caspase^ carrying the K1108A mutation,^23^ only Flag-p40Met^caspase^ showed substantial pro-apoptotic activity, leading to 16% cleaved-caspase-3-positive cells. The respective percentages for p40Met^caspase^ and the K1108A mutant were only ~5 and 2% (Figures 4e and f).

### Calcium stress increases Met shedding, which participates in Met degradation

**Figure 5:**
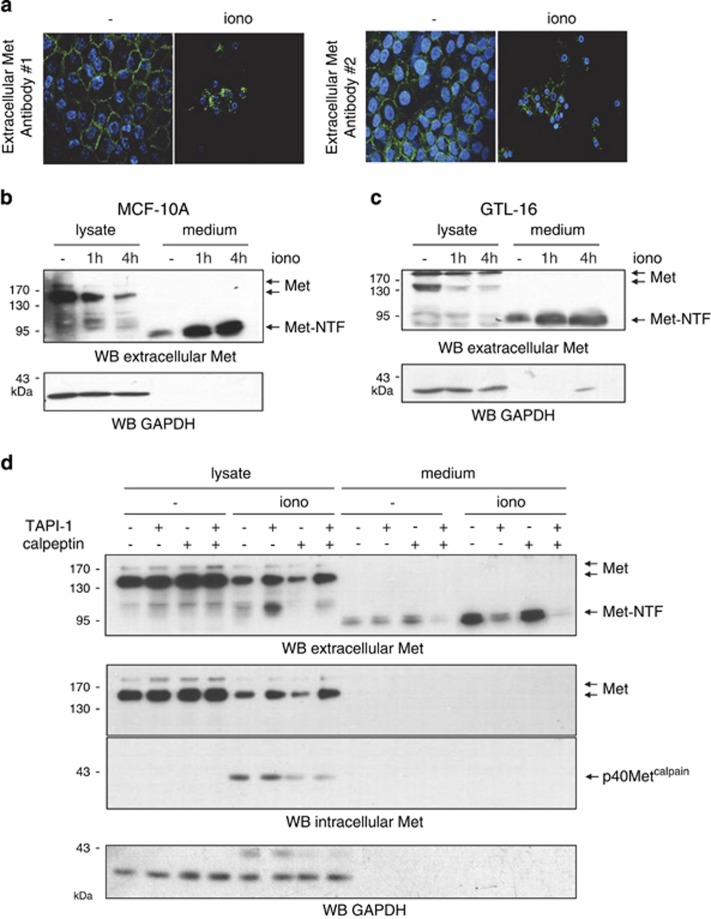
Ionomycin (iono) treatment increases Met shedding. (**a**) MCF-10A cells were grown on glass coverslips, serum starved overnight, and treated with vehicle or 1 *μ*M ionomycin for 4 h. Immunofluorescence staining was performed with two different antibodies directed against the Met extracellular region and the nuclei were stained with Hoechst. (**b**) MCF-10 A and (**c**) GTL-16 cells were grown for 24 h, serum starved overnight, and treated with 1 *μ*M ionomycin for the indicated time. Both cell lysates and an equal volume of conditioned medium were analyzed by western blotting with an antibody against the Met extracellular region. (**d**) MCF-10A and GTL-16 cells were grown for 24 h, serum starved, and pretreated overnight with TAPI-1 and/or calpeptin, and treated for 1 h with 1 *μ*M ionomycin. Cell lysates and conditioned medium were analyzed by western blotting with an antibody against the Met extracellular region, the Met kinase domain (intracellular Met), and GAPDH to assess loading. Arrows indicate positions of full-length Met, Met-NTF, and p40Met^calpain^

We next wondered whether the intracellular cleavage yielding p40Met^calpain^ might also yield a membrane-anchored Met-NTF. Immunostaining with two distinct antibodies failed to reveal any Met-NTF at the membrane surface of MCF-10A cells undergoing necrosis (Figure 5a). Western blotting also failed to reveal the Met-NTF (Figures 5b and c). In contrast, analysis of conditioned medium revealed abundant accumulation of an N-terminal fragment of ~95 kDa (Figures 5b and c). These results suggest that, in addition to calpain processing, Met undergoes an extracellular cleavage releasing its N-terminal region into the medium. According to its apparent size, this N-terminal fragment could be Met-NTF, generated by Met shedding mediated by ADAM metalloproteases during PS-RIP.^18^

The ionomycin-induced appearance of Met-NTF in the conditioned medium was efficiently inhibited by the metalloprotease inhibitor TAPI (TNF-*α* processing inhibitor), but not by the calpain inhibitor calpeptin (Figure 5d). TAPI treatment did not affect p40Met^calpain^ generation, indicating that the two proteolytic processes are independent. Furthermore, in contrast to calpeptin treatment, TAPI treatment was found to rescue full-length Met, indicating that shedding is the major event involved in Met degradation during calcium stress (Figure 5d).

Shedding of the Met-NTF generates a C-terminal counterpart of ~55 kDa (Met-CTF), which is efficiently degraded by the lysosome or further cleaved by *γ*−secretases.^18, 19^ Consistently with this, ionomycin treatment led to increased detection of Met-CTF when this fragment was stabilized by treatment with lysosome and *γ*−secretase inhibitors. This confirms that Met undergoes metalloprotease-mediated shedding during necrosis (Supplementary Figure S4). To assess whether Met-CTF is generated upon receptor cleavage within the extracellular juxtamembrane region, we looked for Met-CTF in MDCK cells expressing either an uncleavable chimeric receptor (uncleavable tropomyosine receptor kinase (TRK)-Met, in which the entire extracellular domain of Met is replaced with the extracellular domain of the TRKA receptor) or a cleavable chimera in which the first 50 juxtamembrane extracellular amino acids of Met are present (cleavable TRK-Met; Supplementary Figure S5A).^18, 19^ As expected, co-treatment with *γ*-secretase and lysosome inhibitors stabilized Met-CTF and the full-length chimera only in cells expressing the cleavable TRK-Met. In these cells, calcium ionophore treatment was found to increase Met-CTF generation, with a concomitant decrease in full-length chimera (Supplementary Figure S5B). In contrast, cells expressing the uncleavable chimera showed no generation of Met-CTF and no degradation of the full-length chimera. Taken together, these data demonstrate that during calcium-stress-induced necrosis, Met is processed by both calpain and metalloproteases, participating in its efficient degradation over a few minutes.

### Met proteolytic processing in lung tumors

**Figure 6:**
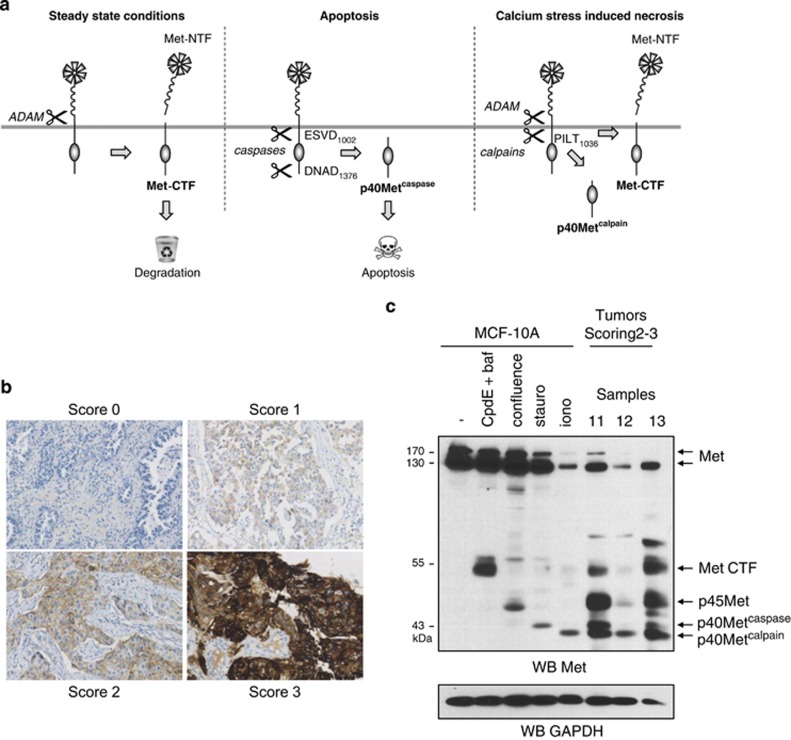
Met fragments are produced in NSCLC tumors overexpressing Met receptor. (**a**) Schematic representation of the different cleavages of the Met receptor. Under steady-state conditions, membrane metalloproteases cause shedding of an N-terminal fragment (Met-NTF) into the extracellular space, with formation of an unstable membrane-anchored C-terminal fragment (Met-CTF). During apoptosis, cleavage of Met by caspases generates p40Met^caspase^, a pro-apoptotic fragment of ~40 kDa. During calcium-stress-induced necrosis, Met is cleaved by both metalloproteases (to Met-NTF and Met-CTF) and calpains (to p40Met^calpain^). (**b**) Tumor samples were analyzed by IHC to determine the level of Met. (**c**) Three tumor samples overexpressing Met were analyzed by western blotting, by comparison with MCF-10A cell lysates treated for 5 h with 1 *μ*M compound E and 5 nM bafilomycin (CpdE+baf), for 6 h with 1 *μ*M staurosporine (stauro), for 1 h with 1 *μ*M ionomycin (iono), or cultured to high density (confluence)

In previous studies and the present one, we have shown that the Met receptor can be cleaved during various physiological processes (Figure 6a).^18, 21^ Although many immunohistochemistry (IHC) studies have shown Met to be overexpressed in a variety of cancers,^14^ the Met cleavage state was never characterized in these studies. About half of all non-small-cell lung cancers (NSCLCs) are known to overexpress Met. We thus analyzed the amount and state of Met by both IHC and western blotting in a library of 13 surgically resected NSCLCs (Supplementary Figure S6A).

Among the tumor samples, four displayed a score of 0, two a score of 1, five a score of 2, and two a score of 3 (Supplementary Figure S6B). Representative Met IHC images are shown (Figure 6b). The amount of full-length Met detected on western blots of tumor lysates was found to correlate, globally, with the Met IHC score (Figure 6c and Supplementary Figure S7). In addition, several Met fragments were detected in tumors with two different antibodies directed against the intracellular region, and their levels were found to correlate with that of full-length receptor (Supplementary Figure S7). Immunoblotting with an antibody directed against the extracellular domain confirmed the full-length Met score and allowed detection of abundant Met-NTFs of ~95 kDa, the likely N-terminal counterparts of the C-terminal fragments.

The main C-terminal fragments observed in tumors, ~55, 45, and 40 kDa in size, are similar in size to the fragments identified in previous studies and the present one. To assess this, we carried out co-migration of three high-Met tumor lysates with cell extracts containing generated or stabilized Met fragments. Met-CTF was stabilized by treatment with *γ*-secretase and lysosome inhibitors, p45 Met was generated in highly confluent cells (manuscript under revision), and p40Met^caspase^ and p40Met^calpain^ were generated by inducing apoptosis or necrosis, respectively, (Figure 6c). The apparent molecular weights of these four fragments perfectly matched those of the Met fragments observed in tumors. It is worth noting that 11 of the 13 tumors had not been subjected to any treatment, suggesting that the Met fragments were not the result of stress induced by therapeutic agents. Unlike the antibodies targeting the Met receptor, an antibody directed against the intracellular domain of epidermal growth factor receptor failed to detect any intracellular fragments. Altogether, these results suggest that proteolytic degradation of the Met receptor is drastically increased in lung cancers overexpressing Met.

## Discussion

Necrosis is a caspase- and ATP-independent cell death process characterized by a loss of plasma membrane and organelle integrity. It lacks features of apoptosis, such as phosphatidylserine externalization and apoptotic body release. Although first defined as accidental cell death, necrosis is currently regarded as a programmed cell death mechanism, as pharmacological inhibitors or gene deletions can protect cells from this demise. Some necrotic cell death can be induced by calcium overload, for example, in neural cells in the case of neurodegenerative disorders or following ischemia.^36^ In the case of cancer cells, cells deficient in Bak (Bcl-2 antagonist/killer) and Bax (Bcl-2-associated X-protein), which are resistant to many apoptosis inducers, have been found to die from necrosis following calcic stress.^33^ Yet the mechanisms underlying calcium-stress-induced necrosis are still poorly understood. As Met is actively involved in both survival and apoptosis, we have sought to determine its fate and potential role during calcium-stress-induced necrosis.

We demonstrate here that calcium-stress-induced necrosis causes a substantial decrease in Met within minutes following treatment with a calcium ionophore. Consistently with this decrease, HGF stimulation can no longer induce Met phosphorylation or activation of downstream signaling pathways under these conditions. This Met degradation might prevent cell protection triggered by ligand-dependent activation of Met. Rapid Met degradation, associated with detection of fragments, suggests that Met undergoes proteolytic cleavages during this process. Accordingly, we have found that Met is cleaved by both calpains and membrane metalloproteases. We show that p40Met^calpain^ generation can be inhibited efficiently by treatment with a pharmacological calpain inhibitor and partially by means of interfering RNAs targeting calpains 1 and 2. This demonstrates that these two proteases, and likely other calpains, are involved in Met cleavage. We demonstrate as well that calcium stress induces autocatalytic cleavage of calpain 1, consistent with activation of calpain by the intracellular calcium level.^37, 38^ *In vitro* cleavage of Met by calpain 1 generates a main fragment of ~40 kDa, confirming the involvement of calpain 1.

Our mass spectrometry and immunoblot analyses reveal juxtamembrane cleavage, possibly at the LT_1036_-S_1037_ sequence, and the presence of the Met C-terminal tail in p40Met^calpain^. Thus, the p40Met^caspase^ and p40Met^calpain^ fragments differ at both their N- and C-terminal ends. Consistently with the fact that p40Met^calpain^ retains the C-terminal tail of Met, known to prevent its pro-apoptotic action,^22^ we show here that it does not induce increased caspase 3 activity and is thus not a pro-apoptotic fragment.

In addition to intracellular p40Met^calpain^, calcium overload induces an increase in two other Met fragments: Met-NTF (in conditioned medium) and the labile Met-CTF, both known to be generated by PS-RIP.^18^ A metalloprotease inhibitor was found to impair production of these fragments and to rescue the full-length Met receptor, demonstrating that calcium stress triggers Met shedding. It is worth noting that inhibition of calpain activity did not rescue full-length Met, suggesting that PS-RIP is the main contributor to the latter's disappearance. The contribution of calpain is hard to assess, as calpain inhibitors only reduced, but did not totally prevent, p40Met^calpain^ generation.

Although Met PS-RIP is mainly involved in Met degradation through generation of labile intracellular fragments, the released Met-NTF is stable and can act as a decoy for HGF/SF.^19, 39, 40, 41^ During calcium-stress-induced necrosis, the amount of released Met-NTF strongly increased, suggesting an increased decoy effect. We thus propose that during calcium-stress-induced necrosis, cleavages decrease the amount of Met receptor and generate a potential extracellular decoy fragment. Interestingly, the N-terminal counterpart of p40Met^caspase^ is a membrane-anchored fragment that can act as a decoy for HGF/SF.^42^ Hence, the stable N-terminal Met fragment generated during apoptosis could be a membrane-anchored decoy, while the fragment generated during calcium stress could be a released decoy, both decoys being able to cause inhibition of an HGF/SF-induced survival response. In the future, it will thus be important to evaluate the decoy role of Met-NTF and its importance in necrosis expansion to the surrounding tissues.

HGF/SF stimulation partially protects cells against calcium-ionophore-induced necrosis and reduces Met cleavage by calpain. Other growth factors have also been shown to protect cells against necrosis, such as the insulin-like growth factor-1, which inhibits calcium-ionophore-induced photoreceptor cell death through expression of the calpain inhibitor calpastatin.^43^ Although the anti-apoptotic properties of HGF/SF have been well described,^44^ further work is needed to elucidate the necrosis-countering pro-survival mechanisms of this ligand.

In NSCLC tumors overexpressing Met, we have identified several C-terminal fragments, including the previously identified Met-CTF (p55 Met), p45 Met generated in highly confluent cells (manuscript under revision), p40Met^caspase^, and p40Met^calpain^. These fragments were mainly detected in tumors showing high levels of full-length Met, except for p40Met^calpain^, detectable in low-scoring tumors. Thus, full-length Met is responsible for only part of the detected signal, as its presence is accompanied by that of multiple C-terminal fragments. This suggests that the level of full-length Met could be overestimated in tumor cells evaluated by IHC. In addition, although the subcellular localization of Met is difficult to establish by IHC, these tumor cells display both plasma membrane and cytoplasmic staining. The latter might be associated with detection of Met C-terminal fragments such as the p40Met^caspase^ and p40Met^calpain^. Consistently, a cytoplasmic and even a nuclear localization of Met have been described in breast cancer samples and mesothelioma tumors.^45, 46^

We demonstrated in cell lines that p40 Met^caspase^ and p40 Met^calpain^ are generated by different molecular mechanisms, and that they do not coexist in the same cell. Because Met fragments produced in NSCLC samples were detected by western blot, which involve the lysis of a cell population, we cannot assert that p40 Met^caspase^ and p40 Met^calpain^ are not produced in the same cells of the tumor. However, it is well established that tumor tissues undergo various stresses influencing differently the cell fates. Thus, the diversity of detected Met fragments might correspond to a diversity of cellular conditions within lung tumors. For instance, the presence of p40Met^caspase^ might be associated with apoptotic cells, whereas the presence of p40Met^calpain^ might be associated with a necrotic area. In these tumor samples, necrotic areas are indeed frequently observed and correlate with a strong decrease of total Met (data not shown). Detection of the labile Met-CTF (p55 Met) in tumor samples suggests that lysosomal degradation is altered in these tumors or that the amount of generated fragment exceeds the capacity of the cells to degrade it. We have demonstrated in a cell model that most of the Met fragments are associated with degradation of the full-length Met receptor. Thus, in tumor samples overexpressing Met, several proteolytic degradation mechanisms might compensate for receptor overexpression, thus limiting the amount of receptor.

At first glance, the mechanism of Met degradation appears similar during apoptosis and calcium-stress-induced necrosis: in both cases, it involves generation of an intracellular fragment of ~40 kDa. In both processes, the extracellular ligand-binding domain of Met is separated from the intracellular kinase domain, preventing survival. Calcium-stress-induced necrosis, however, triggers a more complex proteolytic process, also involving cleavage by membrane metalloproteases. Consequently, the cleavages occurring during necrosis are quicker than caspase cleavages, leading to Met degradation within minutes of stress induction. In addition, while p40Met^caspase^ somewhat resembles p40Met^calpain^, these two fragments appear to be functionally different: the former can favor cell death while the latter is unable to promote such a response.

## Materials and Methods

### Cytokines, drugs, and cell cultures

Ionomycin and A23187 were purchased from Santa Cruz Biotechnology (Dallas, TX, USA). Staurosporine was obtained from Sigma-Aldrich (St Louis, MO, USA). Purified calpain 1 was obtained from Calbiochem (San Diego, CA, USA). Recombinant human HGF was obtained from Peprotech (Rocky Hill, CT, USA). The caspase inhibitor QVD-OPh (*N*-(2-quinolyl)-l-valyl-l-aspartyl-(2,6-difluorophenoxy) methylketone), the calpain inhibitors ALLN (*N*-Acetyl-l-leucyl-l-leucyl-l-norleucinal) and calpeptin, the metalloprotease inhibitor TAPI-1, and the H+-pump inhibitor bafilomycin A1 were purchased from Calbiochem. The cathepsin inhibitor Z-FA-FMK (Z-Phe-Ala fluoromethyl keton) was obtained from Bachem (Budendaurf, Switzerland). The proteasome inhibitor lactacystin was from Sigma-Aldrich. The *γ*-secretase inhibitor compound E was from Alexis/Coger (Lausen, Switzerland).

MDA-MB-231 and GTL-16 cells were cultured in Dulbecco's Modified Eagle's Medium (Life Technologies, Carlsbad, CA, USA) supplemented with 10% fetal bovine serum (FBS; Life Technologies) and 1% antibiotics (penicillin (10 000 U/ml)–streptomycin (10 000 *μ*g/ml); Life Technologies). HEK-293T cells were cultured in DMEM supplemented with 10% FBS, 1% non-essential amino acids (Life Technologies), and antibiotics. MCF-10 A cells were cultured in DMEM and HAM's F12 (vol/vol, Life Technologies) supplemented with 5% horse serum (Life Technologies), 500 ng/ml hydrocortisone (Calbiochem), 20 ng/ml epidermal growth factor (Peprotech), 10 *μ*g/ml insulin (Sigma-Aldrich), 100 ng/ml cholera toxin (Calbiochem), and 1% antibiotics. Calcium-free DMEM was purchased from Life Technologies.

### Antibodies

Rabbit polyclonal antibodies directed against phosphorylated (Y1234/1235) Met (no. 3126), phosphorylated (S473) Akt (no. 9271), and active caspase 3 (no. 9661) and mouse monoclonal antibody directed against phosphorylated (T202/Y204) Erk were purchased from Cell Signaling Technology (Danvers, MA, USA). Mouse monoclonal antibody against the kinase domain of Met (3D4) was purchased from Life Technologies. Mouse monoclonal antibody against glyceralehyde-3-phosphate dehydrogenase (GAPDH; 6C5-32233), rabbit polyclonal antibodies against the C-terminal domain of human PARP-1 (H250), against Akt (H136), and against ERK2 (C14), and goat polyclonal antibodies against the calpain 1 large subunit (C-20) and the calpain 2 large subunit (C-19) were purchased from Santa Cruz Biotechnology. Rabbit monoclonal antibody directed against the C-terminal tail of Met (SP44) was purchased from Roche (Schlieren, Switzerland). Mouse monoclonal antibody directed against the extracellular domain of Met (MAB3582) and goat polyclonal antibody directed against the extracellular domain of Met (AF276) were purchased from R&D Systems (Minneapolis, MN, USA). Mouse monoclonal antibody directed against the C-terminal domain of Met (DL-21) was kindly provided by Dr. Sylvia Giordano (University of Torino Medical School, Italy). Regions recognized by the anti-Met antibodies are shown in the Supplementary Figure S8. Mouse monoclonal antibody directed against Grb2 (growth factor receptor-bound protein 2; 81/GRB2) was purchased from BD Transduction Laboratories (San Jose, CA, USA). Green-fluorescent Alexa Fluor 488-conjugated anti-mouse IgG (H+L) and red-fluorescent Alexa Fluor 594-conjugated anti-rabbit IgG (H+L) were purchased from Life Technologies. Rabbit polyclonal antibodies against the Flag epitope were purchased from Sigma-Aldrich. Rabbit polyclonal antibody directed against tubulin (PM054) was purchased from Medical and Biological Laboratories (Nagoya, Japan).

### Plasmid constructs

The vector expressing Flag-p40Met^caspase^ was constructed as described previously.^22^ The vector expressing p40Met^calpain^ (Met S1037–S1390) was constructed as follows. The portion of Met between D1030 and S1390 was amplified by PCR from pRS2 hu Met (kindly provided by Dr. G Vande Woude, Van Andel Research Institute, MI, USA) used as template, with the following primers 5′-AGGGATCCATGGACATGTCCCCCATCCTAACTAG-3′ containing a *Bam*H1 restriction site and 5′-AGCTCGAGCTATGATGTCTCCCAGAAGGAG-3′ containing a *Xho*1 restriction site. The PCR product was subcloned into pcDNA3 between the *Bam*H1 and *Xho*1 restriction sites. The amino acids D1030–S1037 were removed with the QuikChange site-directed mutagenesis system of Stratagene (Santa Clara, CA, USA), with the following primers: 5′-GTACCGAGCTCGGATCCATGAGTGGGGACTCTGATATATC-3′ and 5′- GATATATCAGAGTCCCCACTCATGGATCCGAGCTCGGTAC-3′. The vector expressing Flag-p40Met^calpain^ was constructed as follows. pcDNA3 p40Met^calpain^ was subcloned into pcDNA3 FLAG between the *Bam*H1 and *Xho*1 restriction sites.

### Transfections and RNA interference

Transfections of HEK-293T and MCF-10 A cells with the reagents polyethyleneamine Exgen 500 (Euromedex, France), and Jet Prime (Polyplus Transfection, Illkirch, FRANCE) were performed as previously described.^22, 23^ For gene silencing, a suspension of 400 000 cells was incubated for 20 min with a mix of 3 *μ*l/ml Lipofectamine 2000 (Invitrogen) and 60 nM small interfering RNA (siRNA), and then plated in a six-well plate in complete medium. The Met-targeting siRNAs were a pool of three stealth siRNAs (Invitrogen) (5′-CCAUUUCAACUGAGUUUGCUGUUAA-3′, 5′-UCCAGAAGAUCAGUUUCCUAAUUCA-3′, and 5′CCGAGGGAAUCAUCAUGAAAGAUUU-3′). The corresponding pool (3 mM) was mixed with control siRNA so as to achieve a total siRNA concentration of 60 nM. The siRNAs targeting calpain 1 and calpain 2 (Sigma-Aldrich) were pools of two siRNAs: calpain 1: (5′-CUAUUGGCUUCGCGGUCUAdTdT-3′ and 5′-GGAACAACGUGGACCCAUAdTdT-3′) and calpain 2: (5′-CGCUAUUCAAGAUAUUUAAdTdT-3′ and 5′-GAAACUGAUCCGCAUCCGAdTdT-3′). Mixtures totaling 60 nM siRNA, containing each pool at 30 nM or one pool at 30 nM and a control siRNA at 30 nM, were prepared.

### Western blotting

Western blotting was performed as previously described.^47^

### Immunofluorescence staining

Cells were grown on glass coverslips for 24 h and then washed, fixed in 4% paraformaldehyde at room temperature for 10 min, and permeabilized in PBS 1 × and 0.5% Triton X-100 at room temperature for 10 min. The cells were then washed and blocked in PBS and 0.2% casein for 30 min. Primary antibodies were incubated for 1 h at room temperature, either alone (mouse anti-extracellular Met antibody (10 *μ*g/ml) or goat anti-extracellular Met antibody (4 *μ*g/ml)) or in combination (anti-Flag antibody (1 *μ*g/ml) and anti-active caspase 3 antibody (1 : 500)). The cells were washed with PBS and incubated for 60 min at room temperature with secondary antibodies diluted to 2 *μ*g/ml. Secondary antibodies were used alone (green-fluorescent Alexa Fluor 488-conjugated anti-goat IgG (H+L), far red-fluorescent Alexa Fluor 647-conjugated anti-rabbit IgG (H+L)) or in combination (green-fluorescent Alexa Fluor 488-conjugated anti-mouse IgG (H+L) and red-fluorescent Alexa Fluor 594-conjugated anti-rabbit IgG (H+L)). The cells were washed with PBS and the nuclei were counterstained with Hoechst 33258. Coverslips were mounted with glycergel mounting medium (Dako, Carpenteria, CA, USA). Slides were observed in oil immersion with an AxioImager Z1 (Carl Zeiss), numerical aperture, ECL-PLAN NEOFLUAR × 40 NA 1.3, with a monochrome Zeiss AxioCam MRm camera and Zen Blue acquisition software.

### Cell death determination

Cells were trypsinized and analyzed with the Tali Apoptosis Kit (Life Technologies). Briefly, cells were harvested by centrifugation and incubated with Alexa Fluor 488-conjugated Annexin V and PI. Then staining was measured with a Tali image-based cytometer (Life Technologies) to determine the amounts of unstained, singly stained, and doubly stained cells.

### *In vitro* calpain cleavage and mass spectrometry

Cells were lysed in lysis and reaction (LR) buffer (100 mM HEPES, pH=7.4, 150 mM NaCl, and 1% Triton X-100). Equal amounts of cell lysate or 5 ng recombinant human Met were incubated at 30 °C with the indicated concentrations of purified calpain 1 and CaCl_2_. The reaction was stopped by adding 3 × Laemmli buffer and heating at 95 °C for 4 min. Samples were analyzed by western blotting.

For mass spectrometry, 900 ng recombinant human Met was incubated at 30 °C for 30 min in LR buffer with 250 *μ*M CaCl_2_ and 2.5 nM purified calpain 1. The reaction was stopped by adding 3 × Laemmli buffer and heating the sample at 95 °C for 4 min. After electrophoresis, the polyacrylamide gel was fixed in 40% methanol, 7% acetic acid solution and stained with Coomassie blue. Stained band corresponding to Met fragment was excised from the gel, reduced, alkylated with iodoacetamide (10 mg/ml in 20 mM NH_4_HCO_3_), and digested overnight with 100 ng AspN endoproteinase (Pierce, Rockford, IL, USA). The resulting peptide mixture was eluted from the gel, desalted, and spotted onto a MALDI plate with freshly dissolved *α*-cyano-4-hydroxycinnaminic acid (10 mg/ml in 50% CH_3_CN, TFA 1/1000). Mass spectrometry was performed by MALDI-TOF-TOF Autoflex Speed (Bruker Daltonics, Fremon, CA, USA). MS and MS/MS data were analyzed with BioTools software (Fremon, CA, USA). Peptides were identified with Mascot, http://www.matrixscience.com/.

### Tumor sample preparation

After surgery, tumor samples were divided into two parts. One was frozen in a Snapfrost fast freezing system (Excilone, Aperio, CA, USA) and stored at −80 °C, and the other was formaldehyde fixed and paraffin embedded (FFPE). For western blot analysis, frozen tumor samples were sliced into pieces ~1.2 mm in diameter and transferred into Lysing Matrix type D tubes containing ceramic beads (MP Biomedicals, Santa Ana, CA, USA) in the presence of RIPA buffer (50 mM Tris-HCl, pH=7.4, 150 mM NaCl, 2 mM EDTA, 50 mM NaF, 0.5% sodium deoxycholate, 0.1% SDS, and 1% NP-40). The samples were lysed with a FastPrep homogenizer (MP Biomedicals; four cycles of 40 s at 6 m/s, each followed by a 5 -min pause on ice). The samples were then centrifuged at 20 000 × *g* for 30 min and proteins in the supernatant were quantified by the BCA Protein Assay (Pierce). Equal amounts of protein were analyzed by western blotting. For IHC, FFPE tissue sections were stained with hematein/eosin/safran and IHC was performed with an antibody against the intracellular domain of Met (SP44 CONFIRM, Ventana Medical Systems). Met expression was scored according to the study of Spigel *et al.*^48^ (score 3: high-intensity staining of at least 50% of the tumor; score 2: moderate staining of at least 50% of the tumor and high-intensity staining of <50% score 1: weak staining of at least 50% of the tumor and moderate or strong staining of <50% score 0: no staining or staining at any intensity of <50% of the tumor).

## Abbreviations

ADAM: A disintegrin and metalloproteinase
ALLN: *N*-Acetyl-l-leucyl-l-leucyl-l-norleucinal
ATP: adenosine triphosphate
Bak: Bcl-2 antagonist/killer
Bax: Bcl-2 associated X-protein
ERK: extracellular signal-regulated kinase
FBS: fetal bovine serum
GAPDH: glyceralehyde-3-phosphate dehydrogenase
Grb2: growth factor receptor-bound protein 2
HGF/SF: hepatocyte growth factor/scatter factor
IHC: immunohistochemistry
Met-CTF: Met C-terminal fragment
Met-NTF: Met N-terminal fragment
NSCLC: non-small-cell lung cancer
PARP: poly(ADP-ribose) polymerase
PI: propidium iodide
PS-RIP: presenilin-regulated intramembrane proteolysis
QVD-OPh: *N*-(2-quinolyl)-l-valyl-l-aspartyl-(2,6-difluorophenoxy) methylketone
RNA: ribonucleic acid
siRNA: small interfering RNA
TAPI: TNF-*α* processing inhibitor
TRK: tropomyosine receptor kinase
Z-FA-FMK: Z-Phe-Ala fluoromethyl ketone
