# Supplementary material for: Necrosis- and apoptosis-related Met cleavages have divergent functional consequences
Source: Cell Death Dis. 2015 May 21;6(5):e1769–. doi: 10.1038/cddis.2015.132 (PMC4669710; doi:10.1038/cddis.2015.132)
Supplement: Supplementary Figures [file cddis2015132x1.pdf]

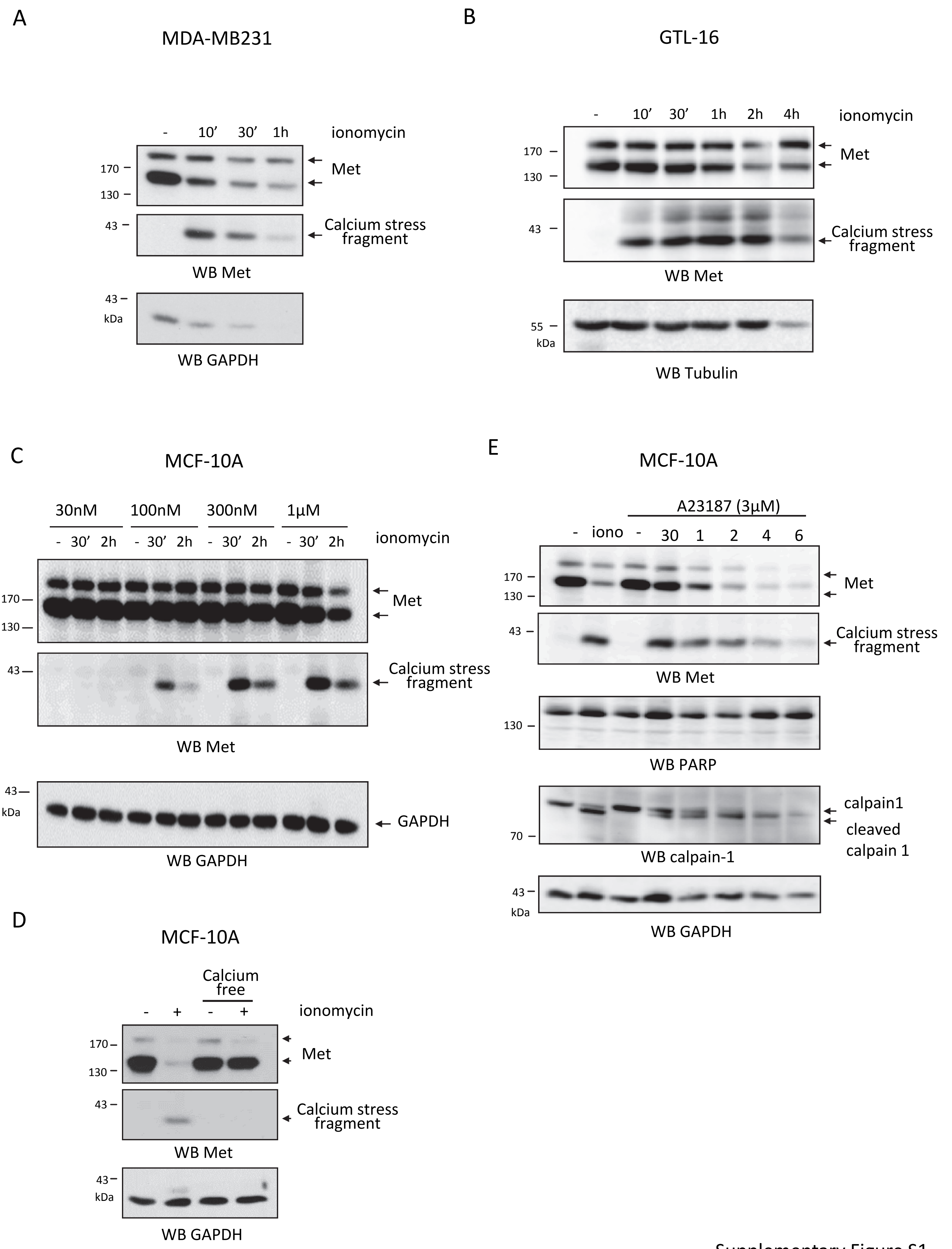

Supplementary Figure S1

A

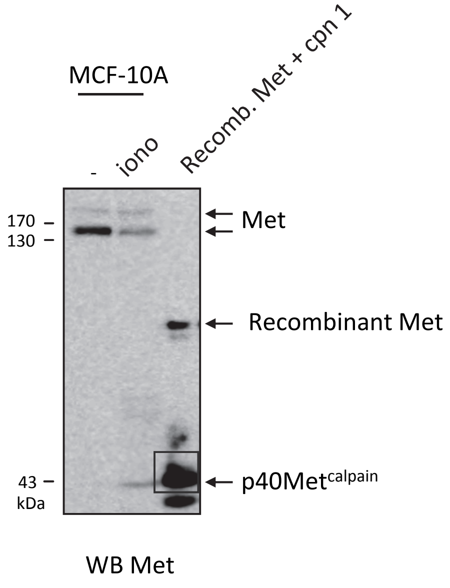

B

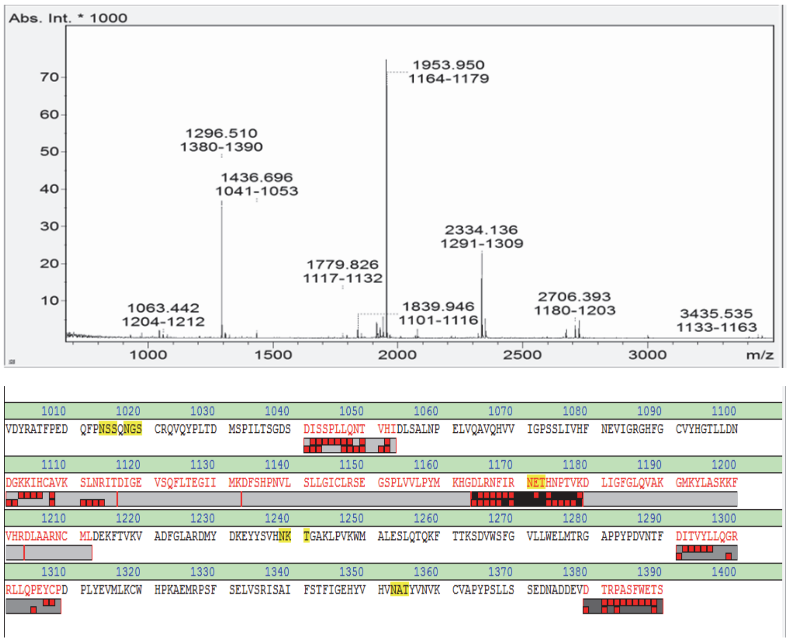

Supplementary Figure S2

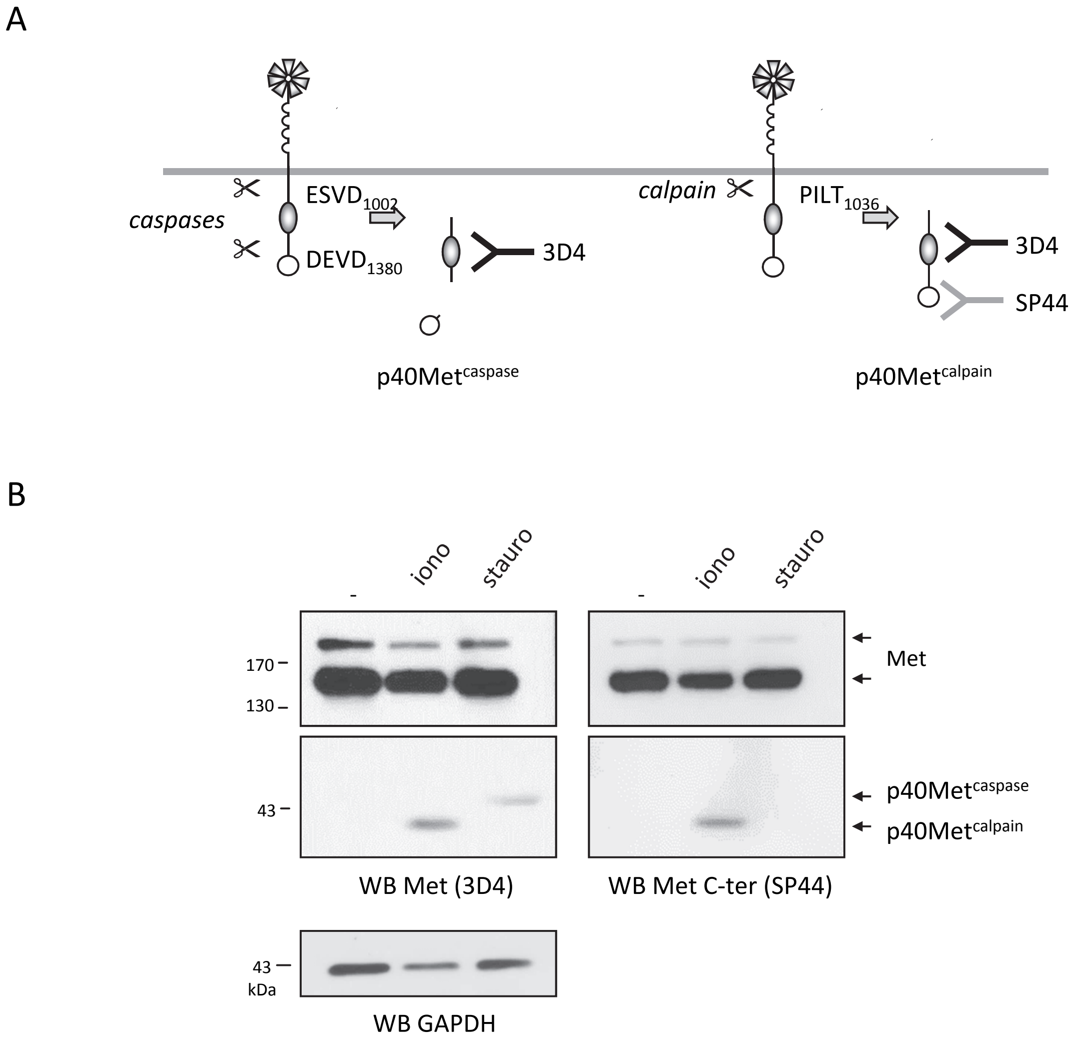

Supplementary Figure S3

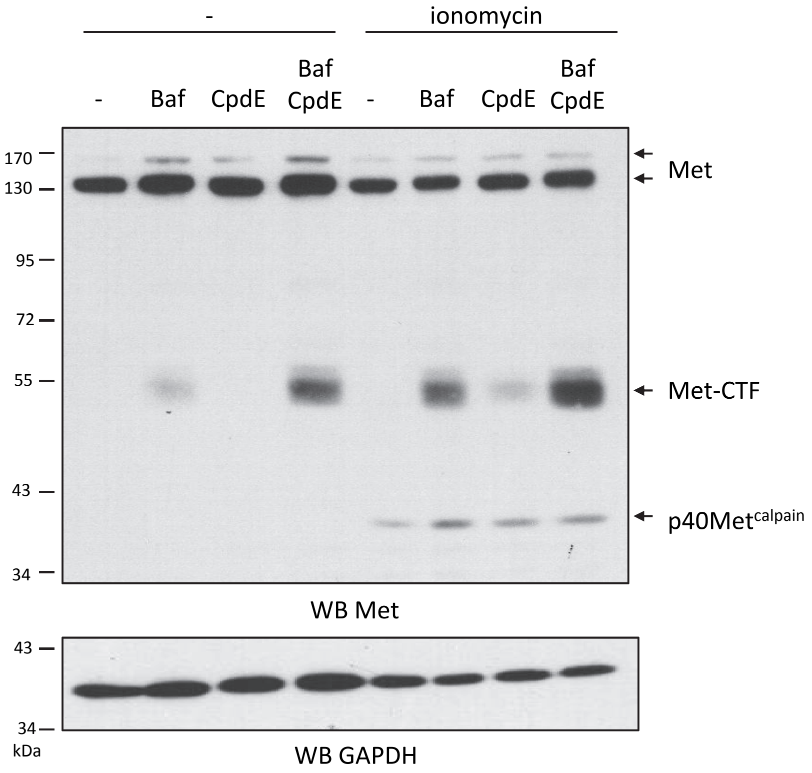

Supplementary Figure S4

A

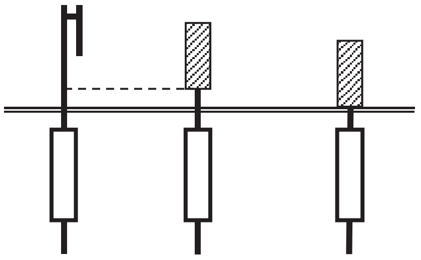

B

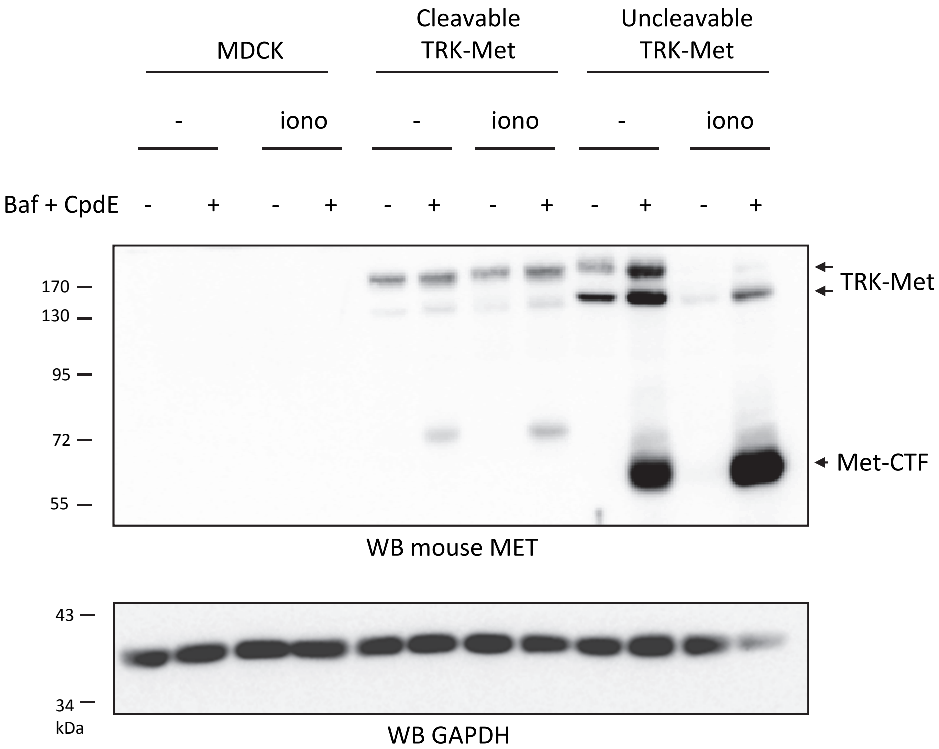

Supplementary Figure S5

A

		n	%
**Age (year)**			
	< 65	10	77
	≥ 65	3	23
**Sex**			
	men	7	53,8
	Women	6	46,2
**Histologic type**			
	Adenocarcinoma	9	69,2
	Squamous cell carcinoma	2	15,4
	Large cell carcinoma	1	7,7
	Large cell endocrine carcinoma	1	7,7
**TNM stage**			
T stage (tumor size)	T1	7	53,8
	T2	4	30,8
	T3	1	7,7
	T4	1	7,7
N stage (Lymph node)	N0	11	84,6
	N1	2	15,4
	N2	0	0
	N3	0	0
	N2	0	0
M stage (metastasis)	M0	13	100
	M1	0	0
**Neoadjuvant chemotherapy**			
	Yes	2	15,4
	No	11	84,6
**WHO clinical stage**			
	0	11	84,6
	1	2	15,4
	2, 3 or 4	0	0
**smoking**			
	former smoker	12	92,3
	smoker	1	7,7
	non-smoker	0	0

B

Sample N°	Histologic type	Met Score (IHC)
1	Adenocarcinoma	0
2	Adenocarcinoma	0
3	Large cell carcinoma	0
4	Large cell neuroendocrine carcinoma	0
5	Squamous cell carcinoma	+
6	Adenocarcinoma	+
7	Adenocarcinoma	++
8	Adenocarcinoma	++
9	Squamous cell carcinoma	++
10	Adenocarcinoma	++
11	Adenocarcinoma	++
12	Adenocarcinoma	+++
13	Adenocarcinoma	+++

Supplementary Figure S6

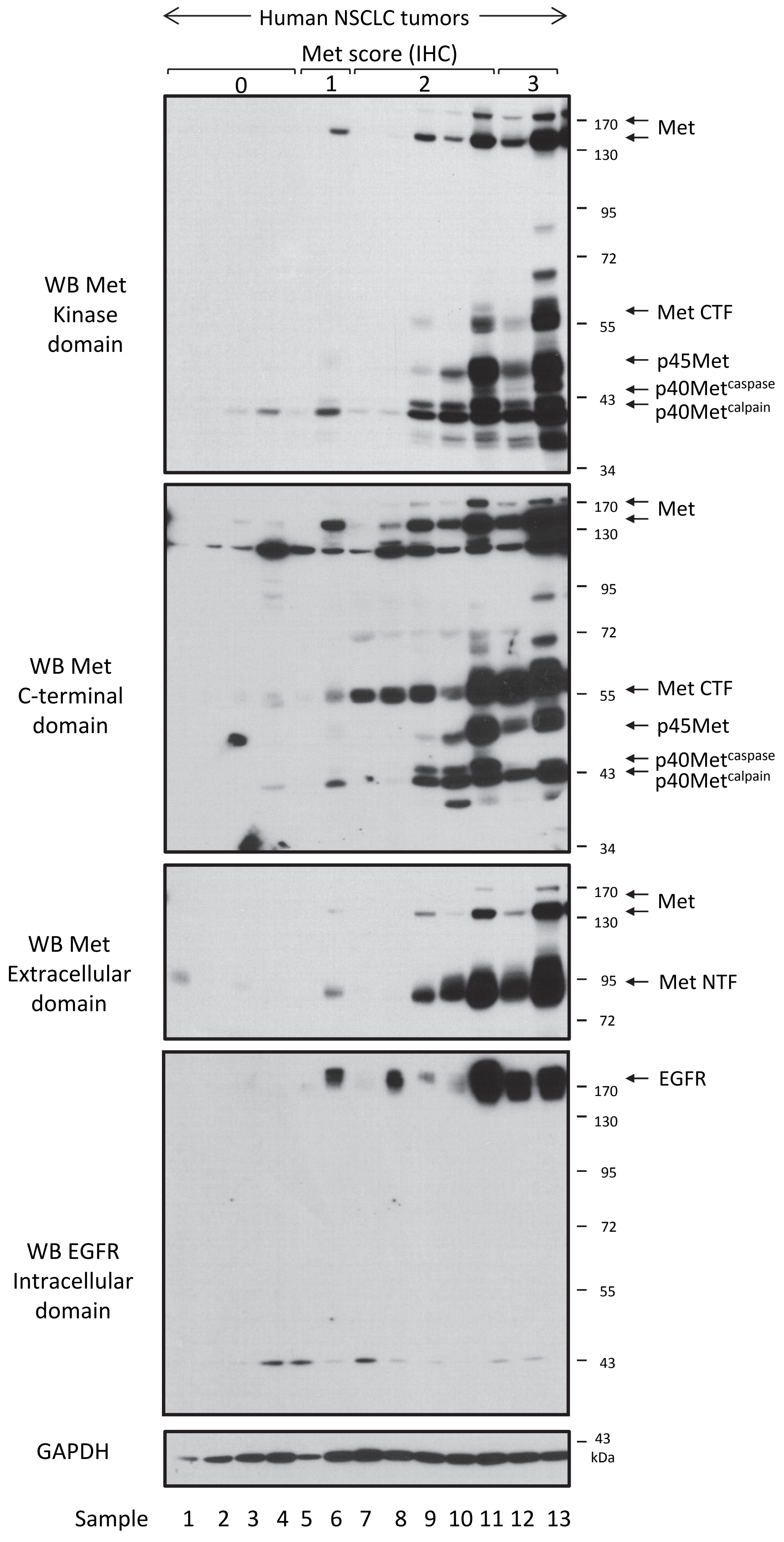

Supplementary Figure S7

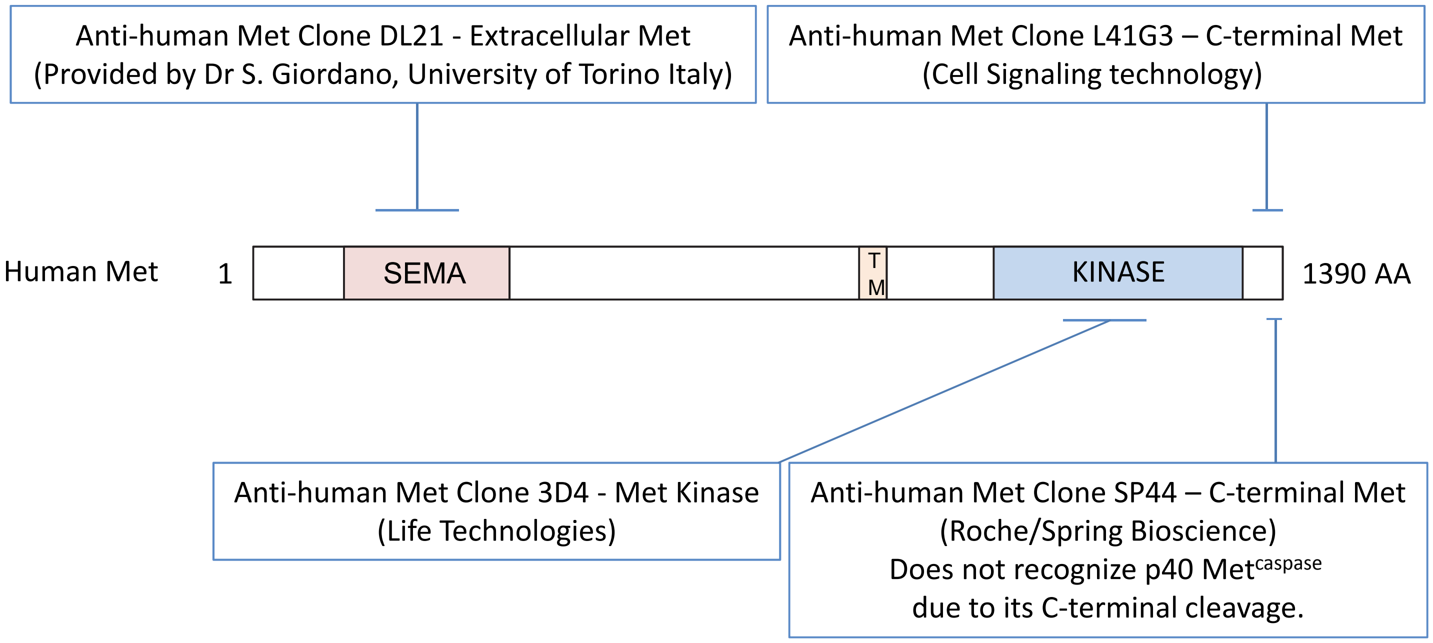

Supplementary Figure S8
