# Supplementary material for: Necrosis- and apoptosis-related Met cleavages have divergent functional consequences
Source: Cell Death Dis. 2015 May 21;6(5):e1769–. doi: 10.1038/cddis.2015.132 (PMC4669710; doi:10.1038/cddis.2015.132)
Supplement: Supplementary Figure Legends [file cddis2015132x2.doc]

**Supplementary Figure Legends**

**Supplementary Figure S1: Induction of Met degradation by A23187 in MCF-10A cells or by ionomycin in MDA-MB231 and GTL-16 cells**

**(A)** MDA-MB231 or **(B)** GLT-16 cells were grown for 24 h, serum starved overnight, and treated with 1 µM ionomycin. **(C, D, E)** MCF-10A cells were grown for 24 h, serum starved overnight, **(C)** treated with 30 nM, 100 nM, 300 nM, or 1 µM ionomycin, **(D)** treated with 1 µM ionomycin after replacing the starvation medium with calcium-free medium, **(E)** treated with 3 µM A23187. Cell lysates were analyzed by western blotting with an antibody against the kinase domain of human Met, against PARP to evaluate caspase activation, against calpain 1, and against GAPDH and tubulin to assess loading. Arrows indicate full-length Met, calcium stress fragment, and full-length and cleaved calpain 1.

**Supplementary Figure S2: Identification of the calpain cleavage site**

**(A)** Recombinant Met was incubated with purified calpain 1 for 30 min. A fraction of the reaction product was allowed to co-migrate with extract of MCF-10A cells treated for 1 h with 1 µM ionomycin. **(B)** After separation of the reaction product by SDS-PAGE, the band corresponding to p40Met^calpain^ was analyzed by mass spectrometry. *Above*: Annotated mass spectrum of AspN digest. *Below*: Protein view, peptides matching the sequence are represented as gray bars, If an MS/MS spectrum is available for the selected peptide, the red bricks inside represent the measured B and Y ions (N- and C-terminal, respectively) matching the peptide sequence.

**Supplementary Figure S3: Calpains do not cleave the Met receptor C-terminal tail**

**(A)** Schematic representation of the cleavage sites of caspases and calpains. Caspases are able to cleave Met at the sites ESVD_1002_ and DNADDEVD_1380_, producing p40Met^caspase^ lacking the last few C-terminal amino acids of Met. We show here that calpains can cleave Met in the juxtamembrane region (PILT_1036_). The 3D4 antibody is directed against the Met kinase domain while the SP44 antibody is directed against its C-terminal tail. **(B)** MCF-10A cells were grown for 24 h, serum starved overnight, and treated with 1 µM staurosporine for 6 h or with 1 µM ionomycin for 1 h. Cell lysates were analyzed by western blotting with 3D4 and SP44 antibodies.

**Supplementary Figure S4: Increased Met-CTF generation during calcium-stress-induced necrosis**

MCF-10A cells were grown for 24 h, serum starved, and pretreated overnight with 5 nM bafilomycin (Baf) and/or 1 µM compound E (CpdE) and treated for 1 h with 1µM ionomycin. Cell lysates were analyzed by western blotting with an antibody against the Met kinase domain and an antibody against GAPDH to assess loading. Arrows indicate the positions of full-length Met, Met-CTF, and p40Met^calpain^.

**Supplementary Figure S5: Calcium-ionophore-induced generation of Met CTF requires the Met extracellular juxtamembrane domain**

**(A)** Schematic representation of full-length Met, uncleavable TRK-Met (consisting of the extracellular portion of TRKA fused to the transmembrane and intracellular domains of Met), and cleavable TRK-Met (possessing 50 additional amino acids of the extracellular juxtamembrane domain of Met). **(B)** MDCK cells expressing the cleavable or uncleavable TRK-Met chimera were grown for 24 h, serum starved and pretreated overnight with 5 nM bafilomycin (Baf) and/or 1 µM compound E (CpdE), and treated for 1 h with 3 µM ionomycin. Cell lysates were analyzed by western blotting with an antibody directed against the C-terminal region of mouse Met and GAPDH to assess loading. Arrows indicate the positions of TRK-Met chimeras and Met-CTF.

**Supplementary Figure S6: (A)** Clinical data pertaining to the 13 NSCLC patients and **(B)** the Met immunohistochemistry scores.

**Supplementary Figure S7: Expression of full-length Met and of its C- and N-terminal fragments in NSCLC tumor samples**

Tumors with scores of 0, 1, 2, and 3 were analyzed by western blotting with antibodies against the Met kinase domain, the Met C-terminal domain, the Met extracellular domain, phosphorylated Met (phospho-Met), the intracellular domain of EGFR, and GAPDH to assess loading.

**Supplementary Figure S8: Schematic representation of Met and the regions recognized by the antibodies used in this study.** The human Met receptor including the SEMA, the transmembrane (TM) and the kinase domain is represented with the approximate regions recognized by four antibodies used for western blot in this study.
